# From Zygote to Blastocyst: Application of Ultrashort Lasers in the Field of Assisted Reproduction and Developmental Biology

**DOI:** 10.3390/diagnostics11101897

**Published:** 2021-10-14

**Authors:** Inna Ilina, Dmitry Sitnikov

**Affiliations:** Joint Institute for High Temperatures of the Russian Academy of Sciences, 125412 Moscow, Russia; sitnik.ds@gmail.com

**Keywords:** femtosecond laser, ultrashort laser pulses, microsurgery, embryo, oocyte, nonlinear microscopy, laser-assisted hatching, embryo biopsy, in vitro fertilization, embryo labeling

## Abstract

Although the use of lasers in medical diagnosis and therapies, as well as in fundamental biomedical research is now almost routine, advanced laser sources and new laser-based methods continue to emerge. Due to the unique ability of ultrashort laser pulses to deposit energy into a microscopic volume in the bulk of a transparent material without disrupting the surrounding tissues, the ultrashort laser-based microsurgery of cells and subcellular components within structurally complex and fragile specimens such as embryos is becoming an important tool in developmental biology and reproductive medicine. In this review, we discuss the mechanisms of ultrashort laser pulse interaction with the matter, advantages of their application for oocyte and preimplantation embryo microsurgery (e.g., for oocyte/blastomere enucleation and embryonic cell fusion), as well as for nonlinear optical microscopy for studying the dynamics of embryonic development and embryo quality assessment. Moreover, we focus on ultrashort laser-based approaches and techniques that are increasingly being applied in the fundamental research and have the potential for successful translation into the IVF (in vitro fertilization) clinics, such as laser-mediated individual embryo labelling and controlled laser-assisted hatching.

## 1. Introduction

It is hard to imagine modern biology and medicine without high-tech equipment, including lasers. Laser radiation in the framework of assisted reproductive technologies (ART) was first applied 30 years ago [1,2] for drilling the zona pellucida (ZP), the outer shell of an embryo. Although the laser pulses were of millisecond duration, their use led to the development of a novel noncontact approach for assisted hatching called laser assisted hatching (LAH). Nowadays, infrared diode lasers are widely applied for laser-induced sperm immobilization and ZP microsurgery, including the aforementioned LAH [2,3], laser-assisted intracytoplasmic sperm injection (ICSI) [4,5], and embryo biopsy [6], turning challenging and time-consuming manipulations into routine procedures.

The evolution of laser technologies has led to a shortening of pulse duration. The term «ultrashort» was introduced and referred to the pulse durations in the femtosecond (10^−15^ s) to picosecond (10^−12^ s) range. Such a pulse is characterized by a high peak power and a broadband optical spectrum (typical bandwidth is about tens of terahertz). Although modern laser facilities are capable of generating pulses with terawatt (10^12^ W) or even petawatt (10^15^ W) peak powers, typical values employed in biological applications lie in kilowatt range. The sources of ultrashort laser pulses have evolved from custom-built laboratory setups to commercial turnkey products as compact as a microwave oven over the past decades. It is the unique properties of ultrashort laser pulses that open up new frontiers in basic research, as well as in various applications. Firstly, due to very short duration of such pulses an investigation of ultrafast phenomena with temporal resolution of femtoseconds can be performed. Secondly, the broad spectral width can be used, for example, in medical diagnostics or for high-precision optical frequency metrology. Moreover, high peak intensity of ultrashort laser pulses may induce nonlinear polarization in media due to first- and second-order nonlinear optical effects including generation of second and third harmonics, optical rectification and two-photon absorption, optical Kerr effect and self-focusing, etc. A comprehensive overview of the effects, which are found in a variety of optical applications can be found elsewhere [7]. When a reversible regime of light-matter interactions is used, various nonlinear microscopy techniques can be realized, while the irreversible regime of light-matter interactions can be applied to nonthermal material processing of a wide variety of materials [8].

An increase in peak intensity has led to the predominance of new mechanisms of laser-matter interaction. Nonlinear optical effects in the focal volume of tightly focused ultrashort laser pulses offer several advantages compared with long-pulsed or continuous wave (CW) lasers. The absence of out-of-focus absorption or significant heat transfer to the surrounding media results in microsurgeries with the crucial properties of superior precision and minimal collateral damage.

In addition to raising the accuracy of microsurgical procedures to a new level, the use of ultrashort laser pulses has created new possibilities for the imaging of cells and tissues. In contrast to conventional microscopy, nonlinear methods have significant advantages such as high resolution and non-invasiveness without the need for exogenous markers.

The aim of this study is to provide an overview of a variety of applications and novel technologies based on ultrashort laser sources of radiation in the field of assisted reproduction and developmental biology. These are not only essential for fundamental research, but also have great potential to be introduced into clinical practice. Technologies of ultrashort laser-based oocyte/blastomere enucleation and embryonic cell fusion as well as advancing ultrashort laser-based technologies for ART, e.g., laser-based individual embryo labelling and controlled LAH at the blastocyst stage are summarized. Moreover, various methods of nonlinear optical microscopy using femtosecond laser pulses for studying the dynamics of embryonic development and embryo quality assessment are discussed. Some applications of ultrashort laser pulses for microsurgery of a variety of externally developing organisms, for instance *Drosophila melanogaster*, zebrafish embryos, etc., are presented in brief.

## 2. Interaction of Femtosecond Laser Pulses with Matter

Let us consider the process of absorption of ultrashort (femto- and picosecond duration) laser pulses in detail. In 1986, it was shown experimentally [9] that the threshold values of optical breakdown for water and biological media were very similar. Some researchers suggested treating the ZP as having the same absorptance and thermal properties as water [10]. This further led to the use of water as a basis for modeling the processes occurring at microsurgical procedures using ultrashort laser pulses. Based on the approach of Williams et al. [11], who proposed to consider water as an “amorphous semiconductor”, in 1991, Sacchi [12] suggested that the band gap (BG) energy separating the valence band (VB) and the conduction bands (CB) was ∆ = 6.5 eV. This approach was widely used to simulate the laser breakdown of water.

**Figure 1:**
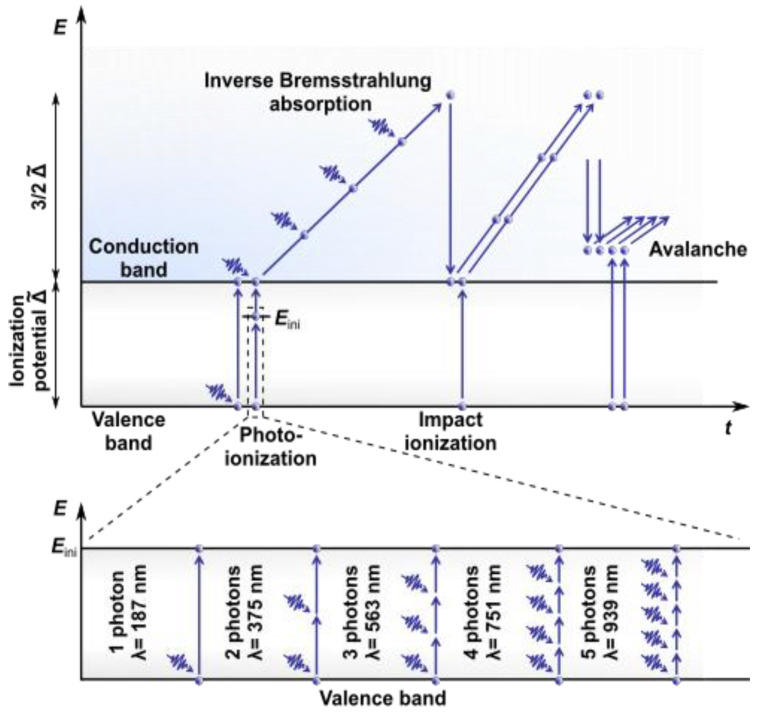
Ionization scheme for laser energy deposition in water.

Modern concepts (Figure 1) assume a more complex energy structure for water. Ionization, the direct transition of an electron to the CB, is considered a two-step process that includes the creation of solvated electrons $e_{aq}^{-}$ at an intermediate level $E_{ini}$ (located 6.6 eV above the upper boundary of the VB) with their subsequent upconversion to the CB (more than 9.5 eV above the VB [13,14]).

### 2.1. Nonlinear Absorption

The absorption of laser radiation in water involves the absorption of photons by electrons accompanied by their transition from the VB to the CB. The energy of a single photon in the visible or near-infrared (NIR) range is insufficient to overcome the BG through both a direct transition and an intermediate level $E_{ini}$ (called “initiation channel” in [15]). It can take simultaneous absorption from three (e.g., for wavelength λ = 514 nm and energy $E_{ph}$ = 2.4 eV) to five (e.g., for λ = 800 nm and $E_{ph}$ = 1.55 eV) photons. Therefore, for ultrashort laser pulses characterized by high intensity, i.e., a high concentration of photons in the focal area, a key role is played by the mechanisms of nonlinear absorption (multiphoton absorption; established in 1931 by Maria Göppert-Mayer in her doctoral thesis [16]) or tunneling ionization. For more details on these mechanisms and their interplay, see [17]. The first electrons in the CB gain kinetic energy through inverse Bremsstrahlung absorption. They can generate further free electrons when their energy exceeds the critical energy required to cause impact ionization [18]. The recurring sequence of inverse Bremsstrahlung absorption events and impact ionization leads to an avalanche growth in the number of free electrons if the irradiance is high enough to overcome the losses of free electrons through diffusion out of the focal volume and through recombination [19]. As a result, the so-called low-density plasma is formed in the focus area of the laser radiation, characterized by the concentration of free electrons $\rho_{c}$. In the case of using long (nanosecond or greater duration) or quasi-CW radiation pulses, the use of dyes that add intermediate energy levels to the BG to make single-photon absorption possible or an increase in the power of laser radiation may be required. This is clearly demonstrated in [20], wherein cell microsurgery using an argon laser at wavelengths λ = 488 nm and λ = 514 nm required a radiation power *P* = 1 W. This value significantly exceeds the average power $P_{av}$ = 30 mW when using femtosecond laser pulse trains at λ = 800 nm [21,22].

### 2.2. Effects Induced

Regardless of the mechanism of electron-hole plasma formation, the absorption of laser radiation in the focus area leads to a temperature increase $\Delta T=\varepsilon /\left(\rho_{0}C_{p}\right)$, determined by the energy density ε absorbed by the plasma, where $\rho_{0}$ and $C_{p}$ are the density and heat capacity of the medium. The energy density of free electrons is defined as the product of the maximum electron concentration $\rho_{max}$ and their average energy $\varepsilon =\rho_{max}\left(\tilde{\Delta }+{\bar{E}}_{kin}\right)$, where $\tilde{\Delta }$ is the effective ionization potential, and ${\bar{E}}_{kin}=(5/4)\tilde{\Delta }$ is the average kinetic energy of free electrons, more details on which can be found in [19]. It has been shown [23] that under water exposure to a series of 170 fs pulses at λ = 800 nm with a repetition rate $f_{rep}$ = 80 MHz and peak intensity $I_{peak}=3.3\times 12^{12}$ W/cm^2^ focused by microobjective with numerical aperture (NA) = 1.3, a temperature of 100 °C can be reached. It is achieved after few microseconds of water irradiation as a result of the dynamic equilibrium between energy deposition and heat diffusion in the focal volume. It should be noted that more than 10^6^ free electrons per pulse are generated; thus, the photothermal effect of denaturation of biomolecules will always be accompanied by free-electron-induced chemical effects, considered below.

The chemical effects in biological media caused by the high reactivity of free electrons are commonly divided into two groups, the first of which comprises changes of the water molecules to create reactive oxygen species (ROS) that subsequently affect organic molecules. OH* and H_2_O_2_ oxygen species have been shown to cause cell damage [24]. The second group comprises direct changes of the organic molecules. The capture of electrons into an antibonding molecular orbital can initiate the fragmentation of biomolecules [19,24,25].

Thermalization of the energy carried by the free electrons, known as an “electron–phonon” interaction, results in a temperature rise in the focal volume on picosecond time scales. This is much shorter than the characteristic thermal diffusion times for water (more than tens of nanoseconds [26]), so the condition for “thermal confinement” is achieved. This local temperature increase in the focal volume results in thermal expansion of the medium. Since energy is delivered faster than the medium can expand, significant transient stresses are developed. As the absorption of photons imparts no significant momentum to the sample, the transient stresses will contain both compressive and tensile components that can produce mechanical damage [19,23,27].

### 2.3. Advantages of Nonlinear Absorption

Multiphoton (nonlinear) absorption is the process of simultaneous capture of *k* photons by the electron. This creates the possibility for transparent materials that do not absorb at a given wavelength at low intensities to display absorption at high intensities when the probability of multiphoton absorption increases. As a result, ultrashort laser pulses can be delivered into a transparent medium and produce interaction in the focal volume only where intensities are high. This is especially important for microsurgery, for example, of an embryo and intracellular organelles, without disturbing the membrane. The nonlinear nature also prevents medium heating along the laser beam path.

The intensity distribution of a laser beam focused by optics with a high numerical aperture has been studied in [28]. It has been shown that spatial distribution of the free electron density is narrower than the irradiance distribution by a factor of $\sqrt{k}$. For λ = 800 nm, it is narrower by $\sqrt{5}=2.24$ and the affected volume is reduced by a factor of 11.2.

Chemical changes, heating, and thermomechanical effects produced by low-density plasmas are thus very well localized because of the nonlinearity of the plasma formation process. They can be produced in a volume smaller than the diffraction limited focus providing higher spatial resolution in terms of microsurgery than linear absorption mechanisms.

## 3. Application of Ultrashort Laser Pulses for Oocyte/Blastomere Enucleation and Embryonic Cell Fusion

As discussed in the previous section, the interaction between laser radiation and biological tissue is a complex process where particular laser-induced effects depend on several values and parameters. Laser radiation parameters such as wavelength, pulse duration, repetition rate, laser power, intensity, and pulse energy should be chosen very carefully, accounting for the characteristic properties of the tissue to be laser-processed. In this section a selection of ultrashort laser-based techniques applied in fundamental research for embryo manipulation including laser-induced embryonic cell enucleation and fusion is presented.

Femtosecond lasers can be used to perform the main steps of somatic cell nuclear transfer (SCNT). The first step in SCNT is called enucleation, followed by the transfer and fusion of a somatic cell into an enucleated oocyte [29]. The action of femtosecond lasers has a significant advantage over other conventional methods such as electrofusion and microinjection with a piezo-manipulator, in that interaction with biological tissues is based on nonlinear absorption, which results in higher penetration depths and absence of out-of-focus absorption and hence reduced (or no) damage to the surrounding organelles and structures. When femtosecond laser pulses are applied for metaphase plate ablation, so-called functional enucleation is performed.

Efficient functional enucleation of mouse oocytes and blastomeres by means of picosecond laser pulses (2 ps duration, 800 nm wavelength, and 80 MHz repetition rate) was shown in [30]. Porcine oocytes were inactivated by means of femtosecond laser pulses (τ = 140 fs/275 fs in the sample, λ = 720 nm, $f_{rep}$ = 1 MHz, *E* = 2.5 nJ, scan speed υ = 100 µm/s) in [31]. Karmenyan et al. reported the optimal conditions for nucleus inactivation in ovulated MII oocytes, activated oocytes, and embryos containing pronuclei and nuclei [30]. Laser-based nucleus inactivation has been demonstrated to be an efficient technique for inactivating the cellular genome without reducing the cytoplasmic volume. The efficiency of functional inactivation was 85% in [30] and 96% in [31], with the maintenance of intact morphology over a long period. The potential for automation of the enucleation procedure by combining femtosecond laser-based metaphase plate ablation with multiphoton microscopy has been demonstrated [31].

Laser-assisted fusion is believed to be an effective noncontact tool for a wide range of biotechnological experiments on gametes and embryos. Cell fusion is used not only for nuclear transfer but also for hybridoma production and reproductive and therapeutic cloning. The successful fusion of two blastomeres at the two-cell stage by application of picosecond laser pulses and generation of viable tetraploid mouse blastocysts has been demonstrated in [30]. The efficiency of cell fusion was shown to be dependent upon the site of laser action and was 35.8% when the ends of the contact border of the blastomeres were exposed to laser irradiation, and 66.7% when the middle region of the blastomere contact border was irradiated with laser pulses.

Successful blastomere fusion using a single femtosecond laser pulse has been demonstrated in [32,33]. For this purpose, the central region of the plasma membrane in the interface between two blastomeres was exposed to femtosecond laser pulses at a wavelength λ = 620 nm (second harmonic of Cr:forsterite laser, τ = 100 fs, $f_{rep}$ = 10 Hz). Cell fusion was usually completed within 20–60 min. The optimal laser exposure parameters for cell fusion were [33]. The highest cell fusion rate (88.9%) was achieved when a single laser pulse with an energy *E* = 35 nJ (fluence *F* = 0.5 J/cm^2^) was applied. About 50% of the fused embryos in this case developed normally up to the blastocyst stage.

It should also be mentioned that the formation of a long-lasting vapor bubble (4–8 µm in diameter) in the irradiated area, creating a pore in both adjacent cell membranes, has been shown to be a prerequisite for successful blastomere fusion [34]. Apart from blastomere fusion, MII-stage oocyte fusion or fusion of oocyte and blastomere has also been demonstrated in [35,36]. For this purpose, the ZP was removed, and lectin was used for cell aggregation. The fusion of plasma membranes took place within 60–90 min of laser treatment.

## 4. Ultrashort Laser Microsurgery of Preimplantation Embryos for Application in ART

There have been several reviews regarding the possible application of lasers in the field of assisted reproduction [37,38,39]. Application of an infrared 1.48 µm diode laser with micro-to-millisecond pulse duration is usually presented. First introduced by Rink et al. [40,41], these laser systems are widely used in in vitro fertilization (IVF) laboratories worldwide. The 1.48 µm diode laser is a compact, easy-to-use device that can be fitted to any microscope and allows for the performance of embryo microsurgery in a contact-free manner. As a rule, laser pulses are applied to create an opening in the outer shell of the oocyte or embryo, i.e., the ZP. The formation of such an opening in the ZP, or laser zona drilling (LZD), is performed in several cases.

**Figure 2:**
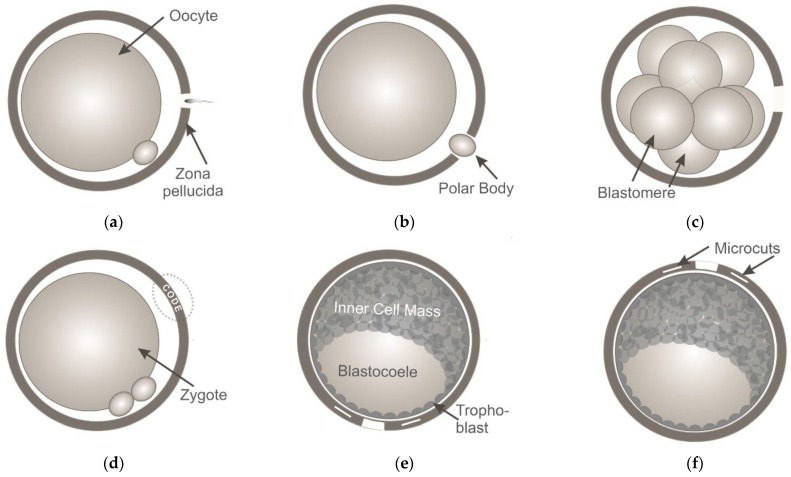

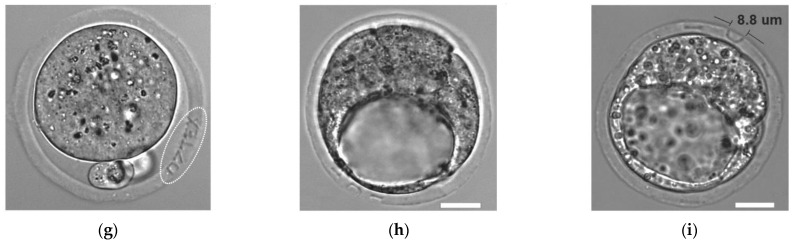
Laser-based ZP microsurgery for applications in ART includes (**a**) in vitro fertilization, (**b**) polar body biopsy, (**c**) laser-assisted hatching; (**d**–**i**) novel techniques based on ZP microsurgery with femtosecond laser pulses (mouse embryos were used in the experiments): (**d**) individual laser-assisted embryo labeling by creating an alphanumerical code on the ZP’s volume and (**g**) example of code “07TEX” engraved on the zygote’s ZP (the dashed circles highlight the codes), (**e**,**f**) controlled laser-assisted hatching at the prescribed location by ZP microsurgery at the blastocyst stage either close to the TE (**e**,**h**) or the ICM (**f**,**i**). Scale bar 20 µm.

First, LZD is believed to be the promising method to assist IVF in humans and mice [41,42,43]. A hole in the ZP (5–10 µm in diameter) is created by applying laser pulses to facilitate sperm penetration and achieve fertilization (Figure 2a). A recent study of Woods et al. has shown that when both male and female subfertility factors exist, laser-assisted IVF reliably ensures a high percentage of two-cell embryos and the production of healthy pups [44]. Infrared diode lasers have been successfully used not only in conventional IVF but also for ICSI—a technique primarily performed for couples with male factor infertility. In laser-assisted ICSI, a small hole is drilled in the oocyte’s ZP while leaving the innermost layer of the zona intact, thus facilitating penetration of the needle with a single sperm though the mechanical barrier with minimal membrane deformation. Laser-assisted ICSI has been shown to result in reduced risk of oocyte damage and oocyte/embryo degeneration, higher rates of fertilization and embryonic development [4,45,46].

The second application of LZD in ART is in preimplantation embryo biopsy. For this purpose, embryonic cells are extracted from the embryo with a glass capillary through a laser-created hole in order to perform preimplantation genetic diagnosis and screening for monogenic diseases or chromosomal abnormalities. In countries where the genetic analysis of embryos is prohibited, polar body biopsy [47] (Figure 2b) is the only method of tissue sampling to screen for maternal mutations. If there are no legal restrictions, embryo biopsy at the cleavage stage (blastomere biopsy [48]) or at later stages of preimplantation development (trophectoderm [TE] biopsy [6]) can be performed.

The third application of LZD is in LAH. Laser-mediated partial thinning or drilling of the ZP (Figure 2c) aims to assist the hatching potential of in vitro cultured embryos and thus improve implantation rates. A systematic review of clinical outcomes following the application of different assisted hatching techniques (chemical, mechanical or laser-assisted hatching) on fresh or frozen/thawed embryos is presented in [49]. Laser-assisted hatching is usually performed on cleavage-stage embryos. Only a limited number of studies has reported performing LAH on blastocysts yet [50,51,52,53,54]. Results from the various studies are very controversial. Discrepancies among studies might be related to variations in assisted hatching techniques and parameters of AH (e.g., irradiation time, time of AH before transfer, size of the hole created, etc.). Literature is consistent in that AH is of no benefit to the overall patient population, however, it might be of value in increasing embryo implantation rates in selected cases. While benefit from ZP breaching or thinning of fresh embryos is still unclear, the situation seems to be different in frozen cycles and a benefit of ZP breaching in thawed blastocysts has been demonstrated. Considering the common use of LAH in IVF laboratories, we believe that the issue regarding the efficacy of the technique will be thoroughly addressed soon, and definitive conclusion will be made. 

Due to the widespread use of infrared diode lasers with milli-to-microsecond pulse duration in ART, numerous attempts have been made to increase their safety and minimize possible adverse effects. While the majority of researchers have noted the relative safety of these laser systems [41,55,56], others have suggested that the heat produced during laser irradiation may have negative effects on embryonic development [57,58,59,60]. To avoid possible laser-related thermal risks, strong recommendations regarding the optimum regimens for embryo exposure have been developed, including requirements for minimizing the pulse lengths in laser devices intended for use in clinical practice or maintaining safe distances between the laser firing position and the nearest blastomere [43,58,60]. Accordingly, most ZP microsurgical procedures are performed at the early stages of preimplantation development when there is sufficient perivitelline space between embryonic cells and the envelope to ensure laser microsurgery at a safe distance.

At the same time, many studies have reported new solutions to issues in assisted reproduction based on laser systems generating laser pulses with shorter durations. For example, successful fully-noncontact polar body biopsy as well as TE biopsy of mouse preimplantation embryos has been demonstrated in [61,62,63] by combining a femtosecond laser (for ZP microsurgery) and optical tweezers (for cell trapping and removal) in a single device. Moreover, unconventional ART procedures can also be performed with femtosecond lasers. In this section, we briefly discuss two novel techniques based on the application of femtosecond laser pulses (Figure 2d–i): a technique for the individual labeling of preimplantation embryos [64,65] and one for controlled LAH [66].

Individual embryo labeling [64] may be useful not only in developmental biology for studying the characteristics of developing embryos during their co-culture in groups, but also in ART for preventing medical accidents relating to the mixing of gametes between patients (usually referred to as “mix-ups”). Although such errors are rare, they periodically occur in IVF laboratories worldwide [67,68]. Such events have serious legal consequences, can cause prolonged emotional distress for the patients, and may directly affect the health of both parents and babies. In order to perform embryo labeling, femtosecond laser pulses with pulse energies of ~20 nJ (second harmonic [λ = 514 nm] of ytterbium laser operating at τ = 280 fs with a repetition rate $f_{rep}$ = 2.5 kHz) were applied to precisely engrave alphanumeric codes in the volume of ZP of mouse embryos at the zygote stage (Figure 2d). The code typically consists of 4–5 characters. An example of an embryo labelled with the code “07TEX” is shown in Figure 2g. To simplify the process of code searching and embryo identification, authors have suggested creating codes not only in a single (equatorial) plane, but in three different planes. There have been no differences in morphology and developmental rates in laser-labelled embryos compared with intact control embryos, and no statistically significant differences in the inner cell mass (ICM):TE ratio between these groups. Importantly, the engraved codes can be clearly recognized until the thinning of the ZP prior to hatching 4.5 days post coitum, enabling embryo identification for nearly the entire period of preimplantation development [65].

As previously mentioned, most ZP microsurgical procedures with milli-to-microsecond laser pulses are performed at the early stages of preimplantation development due to safety concerns. However, it has been shown that premature contact of cleavage-stage embryos with the external environment may be harmful to their subsequent development and may reduce the cell number in the blastocyst when ZP drilling is performed too early during the cleavage stage [69,70]. Thus, ZP drilling at the late stages of preimplantation development may be advantageous compared with early-stage embryo microsurgery. Apart from the fact that the embryo is left undisturbed up to day 5, 6, or even 7 (in humans), ZP drilling at the late stages of preimplantation development (when the ICM and TE are well distinguishable) can be performed at the preferred site and may be beneficial if a TE biopsy is required. ZP drilling close to the TE cells would stimulate embryo hatching in a “trophoblast first” manner, thus making the process of TE biopsy easier. At the same time, Ebner et al. [71] suggested that ZP drilling close to the ICM could result in a developmental advantage due to the acceleration of contact with the endometrium. Thus, if a TE biopsy is not required, ZP drilling at the embryonic pole can be performed to confirm or deny the hypothesis of Ebner et al. However, the technique applied for ZP drilling in this case should provide extreme precision and safety, as the ICM will give rise to the embryonic disk and ultimately the fetus.

Accordingly, Ilina et al. [66] decided to employ the benefits of precise and delicate femtosecond laser-based microsurgery and performed ZP microsurgery of mouse embryos at the late stages of preimplantation development (early blastocyst stage) in order to stimulate embryo hatching to start at a prescribed location. Laser-assisted ZP drilling with femtosecond laser pulses (λ = 514 nm, τ = 280 fs, $f_{rep}$ = 2.5 kHz, *E* = 28.4 ± 1.5 nJ) was performed in two ways: at a point close to the TE cells (Figure 2e,h) and at a point close to the ICM (Figure 2f,i). Additional longitudinal cuts (5–7 μm long) on either side of the hole were created to determine whether hatching had started at the correct location. Data on the hatching start point, as well as embryo viability and preimplantation development in both experimental groups, were collected. A high probability of embryo hatching through the artificial opening (93.3% through the hole created close to the TE and 97.4% through the hole created close to the ICM) was demonstrated. Higher hatching rates in both experimental groups compared with the control groups (96.6% and 97.4% vs. 85.7% and 83.3%) were observed, and the further intrauterine embryonic development of laser-treated embryos was not compromised. Thus, the successful application of femtosecond lasers for embryo microsurgery at the late stages of preimplantation development has been demonstrated.

Summarizing the main points, we may conclude that ultrashort lasers may be considered as an alternative to conventional commercially available infrared diode lasers for application in ART. While the former are more expensive, complex (a certain level of technical expertise is required) and not so compact in size as the latter, ultrashort lasers ensure minimal risk of thermal damage and have the advantage of being multifunctional—they can be used for various applications. Due to nonlinear absorption mechanisms, photon interaction with biological tissue occurs only in the most intense region of the laser focal spot, beyond the diffraction limit, thus making possible to perform embryo microsurgery with high spatial resolution.

## 5. Application of Ultrashort Laser Pulses for Nonlinear Microscopy

In this section, various methods of nonlinear optical (NLO) microscopy using femtosecond laser pulses as well as their advantages over traditional fluorescence microscopy are discussed. The essence of the latter is to register the radiation of light-emitting probes (fluorescent proteins, dye molecules, and semiconductor nanoparticles) chemically associated with specific biological elements (proteins, DNA, phospholipids, etc.). To excite these probes, radiation from a lamp, photodiode, or laser diode in conventional fluorescence microscopy can be used. Spatial distribution of probes after spectral filtering is recorded either in widefield or point scanning mode with subsequent image reconstruction. To improve image quality, equipment such as automated scanners, high numerical aperture lenses, vertical stages, and pinholes is used to achieve spatial filtering and remove out-of-focus light or glare.

Studying the dynamics of embryonic development using classic fluorescence methods can be difficult for several reasons. First, exposure to high-intensity light [72] or even visible light emissions [73,74,75] can damage cells and affect adversely oocytes and embryos. The second reason is the need to monitor the object for a certain period, from several hours to several days. Most of our understanding of dynamic morphological changes is based on the analysis of fixed samples at various stages of development; therefore, the improvement of long-term fluorescence imaging methods is highly necessary. The embryo must be able to undergo cell division during and after imaging. This will provide a better understanding of cell biology and embryonic development including ion dynamics, cytoplasmic reorganization, compaction, and formation of blastocoels [76].

The development of NLO microscopy overcame most of the previous limitations and made it possible to bring the study of intracellular biological processes to a qualitatively new level. For example, multiphoton absorption processes made it possible to avoid staining and obtain rich information on the structural, morphological, and molecular properties of a sample demonstrating a distinctive chemical composition and/or nonlinear properties [77]. The advantages of NLO microscopy also include a high penetration depth due to the use of infrared radiation and a small focal volume that facilitates functional imaging [78,79] as well as effective imaging at greater depths [80].

**Figure 3:**
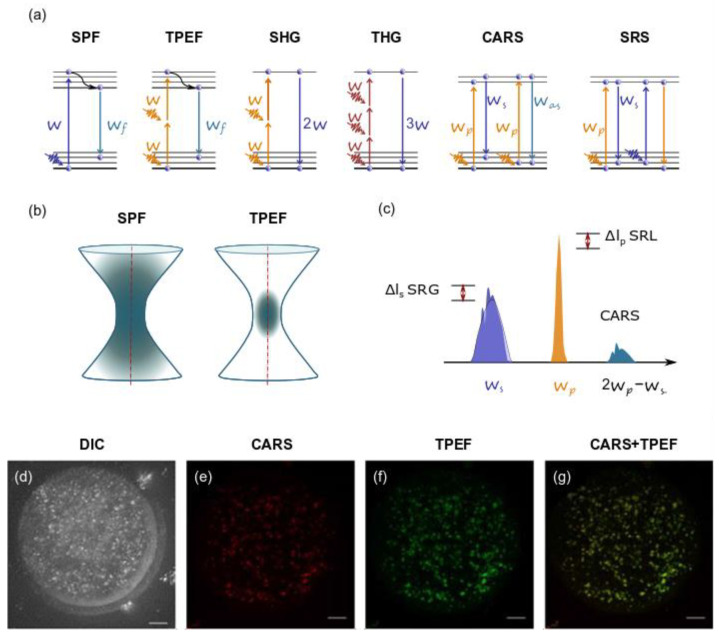
Nonlinear microscopy modalities: (**a**) diagrams (from left to right) for single-photon (SPF) and two-photon excited (TPEF) fluorescence, second harmonic (SHG) and third harmonic (THG) generation, coherent anti-Stokes Raman scattering (CARS) and stimulated Raman scattering (SRS); (**b**) excited volume in case of SPF and TPEF; (**c**) schematic representation of the excitation and emission frequencies involved in SRS and CARS: in evidence the SRS signal in terms of gain (SRG) on the Stokes pulse or the loss (SRL) on the pump pulse. (**a**–**c**) are redrawn from an open-access (CC BY license) source; ref. [77], Authors: Parodi, Jacchetti, Osellame, Cerullo, Polli and Raimondi. Copyright © 2021, Frontiers. (**d**–**g**) An example of CARS and TPEF images of fixed mouse egg: (**d**) DIC image of germinal vesicle mouse egg, (**e**) false-colored CARS image at wavenumber 2850 cm^−1^ and (**f**) TPEF xy image, accompanied by (**g**) false-colored overlays of germinal vesicle egg stained with BODIPY lipid stain. Scale bar: 10 µm. Fragment (**d**–**g**) is adapted with permission from an open-access (CC BY license) source; ref. [86] Copyright © 2021. Published by The Company of Biologists Ltd.

NLO microscopy includes several methods, of which two-photon excited fluorescence (TPEF) [80], generation of the second and third harmonics (SHG and THG) [81,82], and coherent Raman scattering (CRS) microscopy [83] are the most suitable for biological research. The principle of operation (Figure 3) of each of these methods is discussed below.

### 5.1. TPEF Microscopy

TPEF is a nonlinear process based on the simultaneous absorption of two photons, discussed in Section 2.1. It is associated with the transition of an electron from the ground state to an excited state and is possible when the total photon energy exceeds the value of the energy gap between those states. The process is made possible by a high concentration of photons in the waist of the laser beam in a small volume (less than a femtoliter for high numerical aperture objectives). Therefore, there is no out-of-focus light or need for confocal pinhole spatial filters.

Two-photon fluorescence microscopy, also known as two-photon laser scanning microscopy, allows for the visualization of both exogenous (dye molecules, fluorescent proteins such as green fluorescent protein (GFP), red and yellow fluorescent proteins, semiconductor quantum dots) [84,85] and endogenous (nicotinamide adenine dinucleotide phosphate, flavin adenine dinucleotide, and flavoprotein) fluorophores [77]. Since TPEF is based on nonlinear phenomenon, it offers a higher penetration of depth of illumination and true 3D scanning capability, as well as better spatial resolution.

### 5.2. SHG

SHG is a second-order NLO process that involves the interaction of two photons in a nonlinear medium with the generation of a new photon with doubled energy [87]. A special feature of the process is the requirement for the medium to be noncentrosymmetric at the excitation wavelength. The technique is based on the induction of nonlinear polarization in the environment, i.e., the use of endogenous factors. SHG is detected in non-inverted materials such as collagen fibers, myofilaments, astroglial fibers, muscles, and polarized tubulin assemblies such as mitotic spindles. A feature of the SHG signal is the presence of a well-defined polarization. Besides this, the forward SHG signal is more intense for thin samples, and only backscattered photons can be collected from thick specimens [88]. The undoubted advantage of the SHG technique is a significant reduction in the phototoxic effect. The energy absorbed during the generation of a photon of the second harmonic is equal to the energy of that photon. Thus, there is no energetic contribution to the biological object under study. This is also true for the THG technique discussed below. Since there is no need to add exogenous markers, SHG microscopy is considered relatively non-invasive.

### 5.3. THG

This NLO process is of the third order and is usually observed for radiation wavelengths greater than 1200 nm. It is accompanied by the transformation of the energy of three absorbed photons into a photon with triple the energy. The signal is observed at optical heterogeneities only due to a mismatch of refractive indices [89,90], e.g., at the interface between two materials. THG signals are commonly obtained from dense, non-aqueous objects such as lipid droplets and mineralized or absorbing structures [91]. In cells, the main contributors of THG signals are mitochondria [92] and lipid bodies [93,94].

A feature of THG is that generation does not require specific symmetries in the material [95,96]. It also does not require the use of dyes; therefore, dye toxicity issues are eliminated. THG comprises an ideal diagnostic tool that provides unique structural, anatomical, and morphological information from various biological samples [97].

### 5.4. CRS Microscopy

Raman scattering is a technique for molecule/material identification based on the characteristic vibrational spectrum. In spontaneous Raman (SR) microscopy, a monochromatic laser radiation at frequency $\omega_{p}$ (“pump”) excites the molecules to a virtual state, which then relax to the ground state, scattering photons with lower frequency $\omega_{s}$ (“Stokes”) [77]. This scattered light is a characteristic “fingerprint” of the molecule/material, since it is directly related to its structural and molecular properties. Due to the low acquisition rate of the SR method, we will focus on CRS microscopy, in which the Raman signal is generated from a coherent superposition of the molecules in the sample. The sample is irradiated by two synchronized ultrashort laser pulses of different frequencies, the pump $\omega_{p}$ and the Stokes $\omega_{s}$. When their difference, $\Omega =\omega_{p}-\omega_{s}$, matches the vibrational frequency, resonant excitation, and in-phase vibration of all the molecules in the focal volume is observed. This boosts the Raman signal by several orders of magnitude, enabling the shortening of the acquisition time from seconds to milliseconds and minimize the light-toxic effects reported in [98]. The two most widely applied CRS techniques are stimulated Raman scattering (SRS) [99] and coherent anti-Stokes Raman scattering (CARS) [100]. In their review, Parodi et al. [77] compare these techniques and describe their principle of operation as follows. “In CARS, the vibrational coherence is read by a further interaction with the pump beam, generating a coherent radiation at the anti-Stokes frequency $\omega_{aS}=\omega_{p}+\Omega$. In SRS, the coherent interaction with the sample induces stimulated emission from a virtual state of the sample to the investigated vibrational state, resulting in a Stokes-field amplification (Stimulated Raman Gain (SRG)) and a simultaneous pump-field attenuation (Stimulated Raman Loss (SRL))” [77].

### 5.5. Studies

The selection of healthy embryos plays an important role in increasing implantation potential in IVF. Conventional imaging tools have functionality limitations in this regard. Differential interference contrast (DIC) microscopy is unable to indicate spindle fibers due to their slight difference in refractive index with the cytoplasm, while the polar light microscopy is insensitive to the detailed inner structures of embryos and cannot image organelles. Both techniques fail to provide 3D reconstruction due to low depth resolution. Dye staining is required for confocal and TPEF microscopies and is too invasive for IVF applications. With the development of novel microscopy techniques, much attention has been paid to the study of their safety and impact on the object under investigation. Direct comparisons of methods using ultrashort laser pulses with traditional fluorescence microscopy were carried out and recommendations were formulated regarding the design of a new generation of imaging systems [101]. Although a wide variety of species has been investigated, including drosophila [102] and zebrafish embryos [103], we restrict ourselves to a review of studies carried out on mammals in this section (Table 1).

The viability of hamster embryos has been studied in [76] following prolonged exposure to excitation illumination. It was demonstrated that conventional laser scanning confocal microscopy (LSCM, laser intensity *I* = 9·10^3^ W/cm^2^; 8 µs dwell time) inhibited embryo development. None of the two-cell embryos with fluorescently labeled mitochondria imaged over 8 h with LSCM (total exposure = 280 μJ per embryo) reached the morula or blastocyst stage. Inhibition was observed by each of the three wavelengths (514, 532, and 568 nm) used for fluorophore excitation. Many of the LSCM-imaged embryos were unable to undergo even a single division. The authors noted that development of unstained embryos was also inhibited under identical excitation conditions. This suggests that radiation at wavelengths of the visible spectra can directly affect embryos. In contrast to LSCM, two-photon laser scanning microscopy (ultrashort Nd:yttrium lithium fluoride (Nd:YLF) pulsed laser with intensity *I* = 6·10^6^ W/cm^2^ at λ = 1047 nm; 8 µs dwell time) was shown to maintain viability of embryos imaged even for 24 h. The authors suggested that the developmental arrest in LSCM-imaged embryos was caused by the generation of free radicals from the excited fluorophore, which damaged cellular components. Moreover, the reduction of viability in LSCM-imaged embryos was probably due to the formation of hydrogen peroxide, whereas embryos imaged with TPEF were not subjected to the same level of oxidative stress. Thus, NIR TPEF microscopy enables the collection of high-resolution images without affecting developmental potential, even with longer and more frequent image collection.

The relative safety of NIR femtosecond laser pulses was confirmed in [92,101]. The normal development of embryos was demonstrated during 1-day-long discontinuous imaging (λ = 1230 nm, τ = 65 fs, $f_{rep}$ = 76 MHz, *P* = 35 mW) of mouse embryos, revealing the processes of morula compaction and blastocyst formation [101]. SHG images demonstrated central spindles during cytokinesis, whereas THG images indicated lipid droplets, nucleoli, and plasma membranes. Harmonic generation techniques helped to disclose various embryo structures at different stages of development. The authors also described the approaches for long-term imaging, including the optical design and embryo culture methods, in detail.

**Figure 4:**
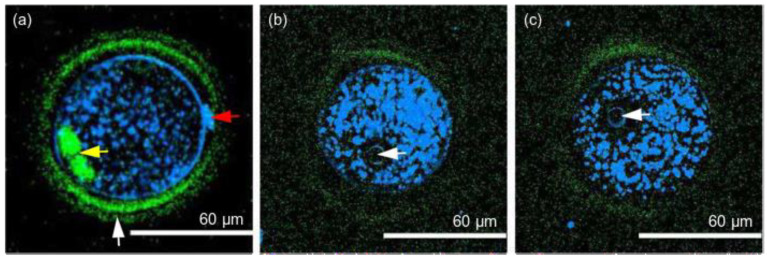
A series of images with combined SHG and THG signals inside the in vitro cultured mouse oocyte: (**a**) the SHG signals reveal the spindle fibers (indicated by the yellow arrow) and the zona pellucida (indicated by the white arrow) of the oocyte, while the THG signals reveal the cell membrane, the organelles, and the polar body (indicated by the red arrow). (**b**,**c**) are images of the same mouse embryo taken at different depths. The white arrows indicate the two pronuclei. The SHG and THG signals are denoted by green and blue colors, respectively. Adapted with permission from [92] © The Optical Society.

The same laser radiation wavelength of 1230 nm was used by Hsieh et al. [92], who demonstrated a 67% development rate of mouse embryos (which was very close to the 70% of the control) after 10 min of continuous imaging using SHG and THG microscopy techniques. The developmental status of the embryos was observed with a high 3D spatial resolution (Figure 4), including the thinning of the ZP, expression of cell adhesion proteins, and cleavage of cells. The authors claimed that THG could provide the contrast required for cell membranes and laminated organelles, including the Golgi apparatus, endoplasmic reticulum, and mitochondria. Blastomeres, the nucleus, and the polar body were also clearly visible. SHG imaging enabled estimation of the thickness of the three layers of the ZP, which is an important factor in selecting proper oocytes. Considering that there is no clinically approved staining dye for embryo and oocyte selection in IVF, Hsieh et al. suggested that these microscopy modalities are an ideal tool to assist in mammalian embryo selection without compromising their viability.

THG microscopy was employed by Kyvelidou et al. to study preimplantation embryo patterning and polarity during the first developmental stages [105]. Femtosecond laser pulses (τ = 200 fs, λ = 1028 nm, $f_{rep}$ = 50 MHz) were applied. The average laser power on the specimen was 20 mW (pulse energy *E* = 0.4 nJ). It took 30 scans to obtain a 2D slice with high signal-to-noise ratio (30 s each) and 10–15 min for 3D THG reconstruction. The THG signal indicated the positioning of mitochondria/lipid bodies. Procedure for quantification of the THG signal was proposed in order to evaluate the THG profile of each developmental stage of the embryo. The highest mitochondrial/lipid body content was observed at the 2-cell stage and decreased thereafter. Moreover, the authors used THG imaging to study blastomere equivalence and observed a divergence of 12–18% of the generated THG signal among blastomeres of the same 8-cell embryo. Such information is believed to be important for selecting the appropriate blastomere for the PDG. Thus, THG imaging was shown to provide valuable information regarding the energetic status of pre-implantation embryos, time evolution of different developmental stages, embryo polarization prior to mitotic division and blastomere equivalence.

Associations between meiotic spindle morphology and oocyte quality have been reported in [109,110,111]. Sanchez et al. [104] suggested that SHG might provide useful information for selecting high-quality oocytes. The ZP and meiotic spindle are the only subcellular structures in mammalian oocytes that produce SHG, with the spindle generating by far the largest signal [104]. To obtain high-quality images of spindles, Ti:sapphire laser (τ = 150 fs, $f_{rep}$ = 80 MHz) with power values of up to 80 mW on the sample was applied for illumination. The authors reported that the SHG method did not significantly impair embryo viability and could be a feasible and safe approach for non-invasive embryo assessment.

THG in combination with phasor fluorescence lifetime imaging microscopy (FLIM) [106] was used to capture endogenous fluorescent biomarkers of preimplantation mouse embryos as a non-morphological marker of embryo quality. The fluorescence lifetime signal form the endogenous molecules was measured to capture data on embryo metabolic states at various developmental stages under normal and nutrient-deficient conditions. At each stage, the mouse embryo displays a characteristic phasor-FLIM signature. THG imaging was employed to characterize the lipid droplets distribution during embryonic development. It was shown that cleavage-stage embryos had a large amount of small, densely packed lipid droplets, whereas post-cleavage-stage embryos had large lipid droplets of low density. Dramatic changes in both lipid oxidation and lipid volume size started after the compaction stages. The authors defined a non-morphological Embryo Viability Index to distinguish pre-implantation embryo quality and demonstrated that the phasor-FLIM approach provides a noninvasive quantitative technology for identifying healthy embryos at the early compaction stage with 86% accuracy.

A significant contribution to the development of non-invasive imaging methods to estimate gamete developmental potential was made in studies [86,108]. Broadband 5-fs Ti:sapphire laser pulses spectrally divided to pump and Stokes beams were applied to demonstrate the differences in the chemical composition of lipid droplets in living mouse oocytes matured in media supplemented with different saturated and unsaturated fatty acids [86]. In a later study [108], CARS imaging was shown to be a non-invasive technique that did not compromise maturation or development in mouse eggs and early embryos. The number, size, and 3D spatial distribution of lipid droplets were examined. Quantitative analysis of their parameters demonstrated statistically significant differences during oocyte maturation and early embryo development. Differences in the size and spatial aggregation of lipid droplets in bovine oocytes compared with mouse oocytes were also observed. The study by Jasensky et al. [107] is also devoted to the assessment and quantification of cytosolic lipid content by CARS, which plays an important role in oocyte development and cryosurvival [112]. Delipation of porcine oocytes by either cytosolic extrusion or removal was shown to significantly improve their survival rate. Quantifiable and consistent differences in percent lipid composition across the oocytes of different species (murine, bovine, and porcine oocytes), developmental stages, and in relation to body composition were observed by the CARS technique. These data suggest that CARS microscopy is a promising technique for assessing specific aspects of the metabolic profile of living mammalian eggs and early embryos, which could be linked to their quality and viability [108].

## 6. Ultrashort Laser Microsurgery of Embryos

This review is mainly focused on the application of ultrashort laser pulses for microsurgery and imaging of preimplantation mammalian embryos. The most suitable and attractive model system in this case is preimplantation mouse embryos. However, several studies report the application of ultrashort laser pulses for microsurgery of a variety of externally developing organisms such as *Drosophila melanogaster* [113,114,115], zebrafish, sea urchin, and starfish embryos [116]. Moreover, the possibility of selective laser ablation of tissues and organs, such as embryonic mouse brains [117] deserves attention. In this section, we will briefly review the results reported in these studies.

Three-dimensional microdissections inside live *Drosophila* embryos (developmental stage 5) with ultrashort laser pulses were performed in [113] in order to locally modify the structural integrity of embryos and thus modulate remote morphogenetic movements. The same laser source was used to perform nonlinear microscopy (TPEF and THG) and track the changes after intravital ablations. The influence of laser microdissections on the process of cellularization has been studied. The photodestruction of a particular region of the embryos has been shown to cause rapid, long-range modulation of morphogenetic movements connected to the targeted area. Such an all-optical approach may find many applications in developmental biology, as it allows for the study of the interplay between cell deformations and molecular signaling.

Hemocyte migration following local injury of Drosophila melanogaster embryos with 100 laser pulses (λ = 355 nm, τ = 470 ps, up to 1 kHz, *E* ~0.74 µJ) was studied in [114] by combining laser nanosurgery with optically sectioning light-sheet based fluorescence microscopy. Following irradiation, GFP-labeled hemocytes moved to the “wounded” area, growing protrusions and potentially removing damaged cells and cellular debris. The authors showed that highly precise, non-contact, three-dimensionally confined plasma-induced laser ablation can be successfully applied in the manipulation of living specimens in 3D.

Experimental investigation and numerical modelling of cell behavior during early gastrulation in *Drosophila* embryos was performed by Rauzi et al. [115]. The authors performed a local tissue immobilization by exposing the apical side of the epithelium to NIR femtosecond laser pulses (λ = 1030 nm, *P_av_* = 200 mW, *t* = 40 ms) and demonstrated interdependencies of epithelial movements during the early stages of gastrulation. The correlation of changes in the behavior and shape of different types of cells with their apical actomyosin architecture and with biomechanical tension was shown.

Controlled damage has been introduced to thick specimens [116] by means of a Ti:sapphire laser integrated within a commercial multiphoton microscope. Laser pulses of ~100 fs duration have been applied for the severing of a dendritic branch of Rohon-Beard neurons within zebrafish embryos, ablation of a mitotic pole within 2-cell-stage urchin embryos, and wounding of the plasma membrane and nuclear envelope within a starfish oocyte. Researchers have demonstrated the lack of toxicity of laser-initiated wounds for the surrounding cells or cytoplasm. The ability to sever neuronal processes at locations that have previously been difficult to reach provides an opportunity to better investigate the mechanisms of axonal degeneration, regeneration, and pathfinding. Moreover, it has been demonstrated that in embryos where the damage was made at the center of a mitotic aster, the first division completed normally; however, the second division failed to complete, which indicated that urchin blastomeres cannot continue normal development without a centrosome. The presence or absence of cytoplasmic changes post laser wounding of the plasma membrane within starfish oocytes has been shown to be dependent on the site where damage was introduced (at the yolk-containing end or clear end). Localized multiphoton wounding of the nuclear envelope was performed to study the requirements for nuclear compartmentation and translocation during meiosis and resulted in nuclear collapse, indicating that loss of the compartmentation barrier makes the structure unstable.

The potential to use ultrashort laser pulses as a histological tool for the precise removal and imaging of brain tissue has been demonstrated by Tsai et al. [117]. The authors accomplished the ablation of fresh and fixed neuronal tissue from adult and embryonic animals and combined it with tissue imaging by two-photon laser scanning microscopy. While amplified ultrashort laser pulses were employed to ablate, unamplified pulses were used to image tissue. The whole process was organized as follows. The surface layers of the tissue were stained if necessary and then imaged. The region of the tissue that was imaged was subsequently removed by laser ablation with ultrashort laser pulses. The newly exposed surface was then re-stained if necessary, imaged, and ablated. To test the capacity for reliable laser-based tissue removal from unfrozen brains, laser ablation and imaging of embryonic mouse brains (embryonic day E15) were performed. Multiple ablation passes at increasing axial depth were performed until over 800 μm of tissue including skin, bone, vasculature, and neuronal tissue was removed. The energy per pulse was set in the range of 23–24 μJ and the scan rate was set to 4.0 mm/s. The authors demonstrated that laser tissue removal preserved optical clarity of the tissue and the ablation process caused no marked distortion of brain structure down to the chromosome level. Thus, the novel technology of all-optical histology, based on the application of laser light to perform both physical sectioning and optical imaging with no need to freeze or embed tissue and register cut sections, has been developed. Most importantly, such a technique may be applied for the analysis of embryonic tissue to study the genetic mutations that produce nonviable animals.

## 7. Conclusions

The rapid development of ultrashort laser technologies has promoted their application in biology and medicine. Due to the unique ability of ultrashort laser pulses to deposit energy into a microscopic volume in the bulk of a transparent material without disrupting the surrounding tissues or tissues above or below the laser focal spot, the femtosecond laser-based modification and ablation of cells and subcellular components within structurally complex and fragile specimens as embryos is becoming an important tool in developmental biology and reproductive medicine. The possible applications of ultrashort laser pulses described in this article are not only limited to ART but also include laser-induced oocyte/blastomere enucleation and embryonic cell fusion—the main steps of SCNT procedure. The application of femtosecond laser pulses has shown promise not only as a microsurgery tool but also as a reliable and convenient tool for precise 3D imaging. As a rule, the very same femtosecond laser system may be used for both cell ablation and imaging. NLO microscopy with femtosecond laser pulses has several advantages over conventional fluorescence microscopy and includes TPEF, generation of the second and third harmonics, and CRS microscopy. NLO microscopy techniques have been successfully applied for embryo visualization, evaluation of their quality and developmental potential, and the study of changes in embryos post femtosecond laser-based microsurgery. Moreover, the application of femtosecond laser pulses is not limited to preimplantation mammalian embryos. In a separate subsection we have briefly discussed the application of femtosecond laser-based microsurgery in externally developing organisms (zebrafish, sea urchin, *Drosophila melanogaster*). With regard to ART, femtosecond lasers have been shown to be an effective and convenient tool to perform not only conventional ART procedures such as embryo biopsy and LAH but also novel techniques such as individual preimplantation embryo labeling and laser-assisted ZP drilling at the blastocyst stage for controlled LAH. As femtosecond lasers offer several important advantages such as high precision, minimal invasiveness, low collateral damage, and versatility over conventional milli/microsecond lasers, we believe that further advances in ultrashort laser technology aimed at reducing the complexity, size, and high cost of femtosecond lasers will help them gain popularity in the field of assisted reproduction.

## Figures and Tables

**Table 1:** NLO microscopy for studying oocytes and embryos.

Object	Method	Parameters	Aim of Study	Results	Source
Two-cell embryos (hamster)	LSCM	Argon laser: λ = 514 nm, *P_av_* = 12 μW, Nd:YAG laser: λ = 532 nm, *P_av_* = 10 μW, Krypton/argon laser: λ = 568 nm, *P_av_* = 28 μW, *t* = 8 μs (dwell time), *E_total_* ~280 μJ per embryo *I_av_* = 9∙10^3^ W/cm^2^	Influence on embryonic development	Confocal imaged embryos never reach the morula stage. Formation of ROS is suggested	[76]
Two-cell embryos (hamster)	Two-photon laser scanning microscopy	Nd:YLF laser τ = 175 fs, λ = 1047 nm, *I_av_* = 6∙10^6^ W/cm^2^, *t* = 8 μs (dwell time), *E_total_* ~2 J per embryo	Influence on embryonic development	Number of embryos developed to morulae and blastocysts did not significantly differ from control ones	[76]
Zygotes-blastocysts (mouse)	SHG, THG	Cr:forsterite laser τ = 65 fs, λ = 1230 nm, $f_{rep}$ = 76 MHz, *P_av_* = 35 mW	Central spindles during cytokinesis (SHG), lipid droplets, nucleoli, and plasma membranes (THG)	Embryos demonstrated normal development during 1-day-long imaging	[101]
Oocytes, embryos (mouse)	SHG, THG	Cr:forsterite laser τ = 140 fs, λ = 1230 nm, $f_{rep}$ = 110 MHz *P_av_* = 150 mW, *E_total_ =* 29 J (per embryo over a total imaging time of 10 min)	Spindle fibers, thickness of the three layers of the zona pellucida (SHG), cell membranes (THG)	67% of embryos have fully developed	[92]
Cryopreserved oocytes and one-cell embryos (mouse)	SHG	Ti:sapphire τ = 150 fs, $f_{rep}$ = 80 MHz, λ = 750 nm, *P_av_* = 3–60 mW; λ = 845/880 nm, *P_av_* = 12–80 mW	Influence of FLIM-based metabolic imaging and SHG spindle imaging on embryonic development	The method does not significantly impair embryo viability	[104]
Zygotes-blastocysts (mouse)	THG	τ = 200 fs, λ = 1028 nm, $f_{rep}$ = 50 MHz, *P_av_* = 20 mW, *E* = 0.4 nJ	Pre-implantation embryo patterning and polarity, blastomere equivalence	THG revealed an energy divergence of blastomeres from 12% to 18%	[105]
Blastocysts (mouse)	FLIM+ THG	Ti:sapphire λ = 740 and 1040 nm, $f_{rep}$ = 80 MHz, *P_av_* = 3.5–15 mW	Developmental states, metabolic changes	FLIM does not disrupt embryonic development under 10 mW. Method for embryo quality estimation and viability prediction is proposed.	[106]
Oocytes (mouse)	CARS	Ti:sapphire τ = 150 fs, λ = 800 nm, $f_{rep}$ = 80 MHz, *P_av_* ~6 mW, t ~2 min (CARS-PMT), t ~20 min (CARS-CCD)	Lipid content in mammalian oocytes during development and in relation to body composition	Quantifiable difference is found in percent lipid composition across oocytes of different species, developmental stages, and in relation to body composition	[107]
Embryos (mouse)	CARS, TPEF	τ = 5 fs, λ = 660–730 nm (pump), λ = 730–900 nm (Stokes), λ = 930 nm (TPEF), *t* = 10 µs (dwell time), $P_{pump}$ = 14 mW, $P_{Stokes}$ = 9 mW	The number, size, and 3D spatial distribution of lipid droplets	The differences in the chemical composition of lipid droplets in living oocytes matured in media supplemented with different saturated and unsaturated fatty acids were observed	[86]
Oocytes, embryos (mouse, bovine)	CARS	τ = 5 fs, λ = 660–730 nm (pump), λ = 730–900 nm (Stokes), *t* = 10 µs (dwell time), $P_{pump}$ = 27 mW, $P_{Stokes}$ = 13 mW	The number, size, and 3D spatial distribution of lipid droplets	Specific aspects of the metabolic profile of living mammalian eggs and early embryos can be assessed	[108]
